# Thermodynamic and Structural Analysis of DNA Damage Architectures Related to Replication

**DOI:** 10.1155/2013/867957

**Published:** 2013-04-28

**Authors:** Nicholas J. Amato, Christopher N. Mwai, Timothy C. Mueser, Amanda C. Bryant-Friedrich

**Affiliations:** ^1^Department of Chemistry, College of Natural Sciences and Mathematics, The University of Toledo, Toledo, OH 43606-3390, USA; ^2^Department of Medicinal and Biological Chemistry, College of Pharmacy and Pharmaceutical Sciences, The University of Toledo, Toledo, OH 43606-3390, USA

## Abstract

Damaged DNA, generated by the abstraction of one of five hydrogen atoms from the 2′-deoxyribose ring of the nucleic acid, can contain a variety of lesions, some of which compromise physiological processes. Recently, DNA damage, resulting from the formation of a C3′-thymidinyl radical in DNA oligomers, was found to be dependent on nucleic acid structure. Architectures relevant to DNA replication were observed to generate larger amounts of strand-break and 1-(2′-deoxy-**β**-D-*threo*-pentofuranosyl)thymidine formation than that observed for duplex DNA. To understand how this damage can affect the integrity of DNA, the impact of C3′-thymidinyl radical derived lesions on DNA stability and structure was characterized using biophysical methods. DNA architectures evaluated include duplex DNA (dsDNA), single 3′ or 5′-overhangs (OvHgs), and forks. Thermal melting analysis and differential scanning calorimetry measurements indicate that an individual 3′-OvHg is more destabilizing than a 5′-OvHg. The presence of a terminal 3′ or 5′ phosphate decreases the Δ*G*_25_ to the same extent, while the effect of the phosphate at the ss-dsDNA junction of OvHgs is dependent on sequence. Additionally, the effect of 1-(2′-deoxy-**β**-D-*threo*-pentofuranosyl)thymidine is found to depend on DNA architecture and proximity to the 3′ end of the damaged strand.

## 1. Introduction

Maintaining DNA integrity is essential to facilitate proper physiological processes including DNA replication, repair, and transcription. The disruption of replication by reactive oxygen species (ROS) has been linked to a variety of diseases and disorders [1–3]. Causative in the development of disease *via* this mechanism is the stalling or collapse of replication forks. When this occurs, atypical secondary structures form which must be resolved and/or repaired before replication can resume [4]. Additionally, it has been shown that elevated levels of oxidative stress during replication result in a prolonged S-phase and if left unrepaired, can lead to apoptosis [5]. 

**Figure 1:**
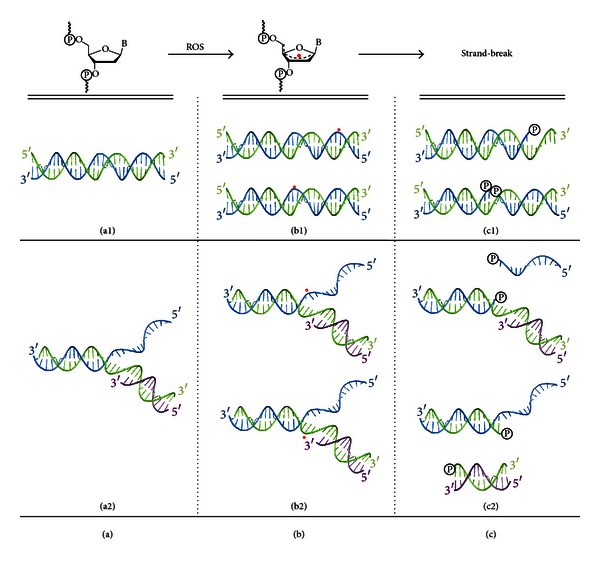
Hydrogen atom abstraction at a 2′-deoxyribose moiety in DNA replication relevant architectures. (a) Model replication substrates: duplex (a1) and flap (a2). (b) 2′-Deoxyribose radical intermediates in duplex (b1) and flap substrates (b2). (c) Strand-break products resulting from oxidative sugar damage in duplex (c1) and flap substrates (c2).

Double strand-breaks (DSBs), being one of the most lethal DNA damaging events resulting from ROS, are known to form through oxidative damage and have recently been reported to be replication induced [6]. Replication induced DSBs are proposed to result from a single strand-break (SSB) and/or other damage lesions generated in replicating DNA [6]. Under conditions of oxidative stress, SSBs can result from hydrogen atom abstraction at the 2′-deoxyribose ring in oligonucleotides. When a single hydrogen atom is removed by a ROS from any of the five carbons of the sugar moiety (Figure 1(a)), a highly reactive sugar radical is produced (Figure 1(b)). When trapping of the radical by a hydrogen atom donor does not occur, a strand-break is usually generated (Figure 1(c)). It is believed that the above-mentioned creation of stalled and collapsed replication forks is related to such sugar damaging events. The resulting substrates then signal spontaneous homologous recombination [7] and nonhomologous end-joining [8]. 

**Figure 2:**
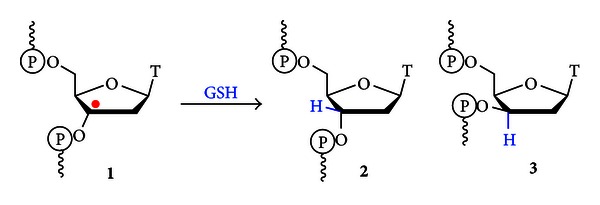
Reduction of the C3′-thymidinyl radical by GSH. Formation of substrates containing the repaired 2′-deoxyribose (**2**) and the 1-(2′-deoxy-*β*-D-*threo*-pentofuranosyl)thymidine (**3**) damage lesion were observed [9].

Our laboratory is interested in determining the role of DNA structure in preserving genomic integrity through protection of the nucleic acid from oxidative damage. We have observed that the selective generation of radical intermediates in DNA architectures that act as models of substrates which form during the process of replication results in elevated levels of strand-break products when compared to duplex DNA. The formation of 3′ and 5′-overhangs (OvHgs) containing a phosphate at the ss-dsDNA junction, as well as duplex substrates containing a terminal phosphate was observed [9]. Additionally, the formation of 1-(2′-deoxy-*β*-D-*threo*-pentofuranosyl)thymidine (2′-deoxyxylothymidine, **3**, Figure 2) was observed as a result of trapping of the C3′-thymidinyl radical from the alpha face of the nucleotide by glutathione (GSH). This damage product was also formed readily in replication relevant architectures, while nearly absent in duplex DNA [9]. Together, these results highlight the protection against oxidative damage offered by the duplex structure of DNA when the C3′-thymidinyl radical is the reactive intermediate [9]. 

The effect of oxidatively generated damage lesions on the structure and stability of DNA substrates has received limited attention [10, 11]. Herein, we analyze the impact of damage lesions on the structure and stability of DNA architectures, which model those generated during replication. The impact of the presence of these lesions on replication derived model architectures reveals how DNA damage alters the stability of nucleotide substrates associated with replication.

## 2. Materials and Methods

### 2.1. Oligonucleotides

Oligonucleotides containing **3** (Z) were synthesized, using established methods, on an ABI 391 automated DNA synthesizer [12]. The synthesis of Sequence 1: 5′-TTTTTZTTTCAGGTTGCG-3′ and Sequence 2: 5′-GCGTTGGACTTTZTTTTT-3′ was performed on a 0.2 *μ*m scale using polystyrene columns (Glen Research, Sterling, VA). Oligonucleotides were cleaved from the resin and the nucleobase and phosphate protecting groups removed by heating at 55°C overnight in aqueous ammonium hydroxide (28–30%). Oligonucleotide purification was accomplished using oligonucleotide purification cartridges (OPC, Glen Research, Sterling, VA) and the samples were quantified (*ε* = 158,100 L/mole∗cm) using UV absorbance at 260 nm on an Agilent 8453 UV-Visible Spectrophotometer. Sample purity was assessed by tandem ion-exchange/reverse-phase high-performance liquid chromatography and the identity confirmed using matrix-assisted laser desorption/ionization time-of-flight mass spectrometry (MALDI-TOF MS). All unmodified oligonucleotides were either synthesized or purchased (Integrated DNA Technologies, Coralville, IA, USA).

### 2.2. UV Melting

Melting experiments were conducted on a Varian Cary 3E UV-Vis spectrophotometer equipped with a temperature controller. Absorbance changes for oligonucleotides (2 *μ*M) in 25 mM chelex-100 treated phosphate buffer (pH 7.3) containing 60 mM potassium acetate and 6 mM magnesium acetate were recorded as a function of temperature from 10–80°C at a rate of 0.5°C/min at 260 nm. Each measurement was performed in triplicate. The average of three scans was taken and reported errors were calculated using standard deviation. The *T*_*m*_ was obtained using the first derivative method. Calculated Δ*T*_*m*_ was determined by subtracting the experimental values obtained for the 2′-deoxyribose containing construct from the corresponding constructs containing **3**.

### 2.3. Circular Dichroism

The circular dichroism spectra for all architectures reported were collected using an AVIV 62DS CD Spectrometer at 25°C, using a quartz cuvette with a path length of 1 mm in a thermostatted holder. Spectra were collected from 320 to 200 nm at a rate of 20 nm/min using a bandwidth of 1 nm. Four scans were accumulated and averaged. Oligonucleotides were measured in phosphate buffer (pH 7.3) at (5 *μ*M). Raw data (Θ) were converted to molar ellipticity ([Θ]) ([Θ] = Θ /(10∗C∗l)) and smoothed using a moving average of three. Cells were cleaned and dried between sample runs according to previously established methods [13]. 

### 2.4. Differential Scanning Calorimetry

Calorimetric excess heat capacity (Δ*C*_*p*_) versus temperature (*T*) profiles was plotted for each DNA architecture using data obtained on a VP Micro-Calorimeter following established methods [14]. Concentration of oligonucleotide samples was 0.2 mM in phosphate buffer (pH 7.3). All samples were stirred and degassed using a MicroCal ThermoVac for 16 minutes at 25°C. Samples were loaded and scanned at a rate of 1°C/min from 10°C to 100°C. Three scans per sample were collected and analyzed using Origin software. Values for Δ*H*_DSC_, Δ*S*, and  Δ*H*_*νH*_ were determined utilizing the equations listed below [15]:

(1) ΔHDSC=∫T0TuCp(ex)dT,ΔS=∫T0TuCp(ex)TmdT,ΔHνH=4RTm2Cp(m)ΔHDSC,

where *C*_*p*_(ex) is the excess heat capacity, Δ*H*_DSC_ is the enthalpy obtained directly from the raw data that has been baseline corrected and normalized, Δ*S* is the entropy calculated without assuming a two-state melting transition, Δ*H*_*νH*_ is the calculated van't Hoff enthalpy, and *C*_*p*_(*m*) is the transition maximum value for *C*_*p*_(ex) [15]. Δ*G* at 25°C was obtained using the following Gibbs free energy equation:

(2) ΔG25=ΔHDSC−TΔS.

Calculations for ΔΔ*H*_DSC_, ΔΔ*S*, and ΔΔ*G*_25_ were obtained by subtracting the values obtained for the core sequence from the corresponding products of interest. Reported errors were calculated using standard deviation. 

## 3. Results and Discussion

### 3.1. Design of Architectures

During DNA replication, both the leading and lagging strand templates undergo a variety of architectural changes including the formation and unwinding of duplexes, as well as the transient formation of overhangs, flaps, and forks. Our previous work as well as that of others [9, 16, 17] has shown that the structure of DNA has a profound impact on the fate of 2′-deoxyribose radicals. After determining the structures of the lesions formed through independent generation of the C3′-thymidinyl radical, we investigated their impact on the structure and stability of DNA architectures that form *via* this type of damage. The interplay between DNA structure and damage outcomes is an important paradigm in our understanding of the ability of DNA to protect itself from damage events and the ability to repair damage when it occurs. The substrates utilized in these studies were designed to allow for the evaluation of the effects of both **3** and the strand-break products observed in our previous work. 

**Figure 3:**
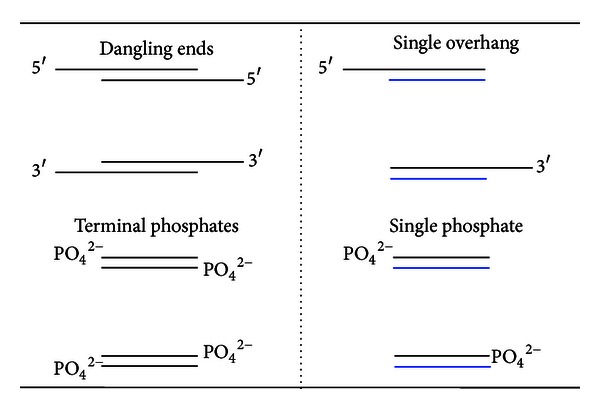
Structural differences in the design of some previously used substrates (left) and herein (right) in characterizing the thermodynamic contribution of a single-stranded DNA region or terminal phosphate (PO_4_^2−^) on the stability of the core dsDNA. Oligonucleotides designed for dangling ends and terminal phosphates are self-complementary, while the evaluation of a single overhang or phosphate does not require self-complementary sequences.

 Generally, the presence of short overhangs or terminal phosphates increases the stability of DNA constructs when compared to the corresponding blunt-ended duplexes [18–22]. In previous work, the effect of overhangs, otherwise known as “dangling ends,” on duplex stability was determined utilizing self-complimentary DNAs that produce a “dangling end” at both ends of the substrate (Figure 3). This design requires consideration of the thermodynamic contribution of two overhangs to DNA stability, which was compensated for by either halving the values of ΔΔ*G* [20] or accounting for the changes induced by both [19, 21]. It is our hypothesis that the presence of “dangling ends” on both ends of the core DNA sequence has an influence on the *T*_*m*_ and/or thermodynamics of melting. This is supported by the results reported in Benight et al. who used substrates containing both a 5′ and 3′-OvHg as well as dangling ends containing two 5′-OvHgs. These substrates indicated that the thermodynamic impact of the overhang was dependent upon the DNA substrates used [23]. The substrates utilized in these studies were designed to allow for the direct evaluation of the effects introduced by a single overhang, phosphate or **3** [9]. 

### 3.2. Stability Studies of Architectures Containing **3**

The melting temperatures for duplex, 5′ and 3′-OvHgs and fork architectures were determined for unmodified architectures as well as those containing **3**. Incorporation of **3** into these architectures revealed that the replacement of a single 2′-deoxyribose with **3** decreases the stability of the duplex region (Table 1). Previously, it was shown that DNA duplexes containing **3** in a single location demonstrate decreased stability when compared to the unmodified duplex. This indicates that the inversion of configuration at the C3′-position of 2′-deoxyribose to 2′-deoxyxylose at a single nucleoside significantly destabilizes the DNA duplex [10, 24]. We made the same observation in the case of DNA duplexes **1D** and **2D**, obtaining decreases in *T*_*m*_ of 5.1°C and 5.3°C, respectively. However, when **3** is found at an overhang or fork junction, the effect is significantly decreased. Melting temperatures of 5′-OvHgs **1A** and **1B** and fork **1E** & **1F** show no appreciable change when **3** is placed at the junction. Alternatively, the 3′-OvHg **2B** and fork **2F** containing **3** at the same position demonstrate a decrease in *T*_*m*_ of 1.4°C and 0.7°C, respectively. As indicated by the respective melting temperatures of **2B** and **2F**, the overall effect of **3** appears to have less of an impact on stability in less stable substrates. These results suggest that the destabilizing effect of **3** is not only dependent on DNA architecture, but also on the location of the modification relative to the 5′ and 3′ ends of the oligonucleotide.

### 3.3. CD Analysis of Architectures Containing **3**

**Figure 4:**
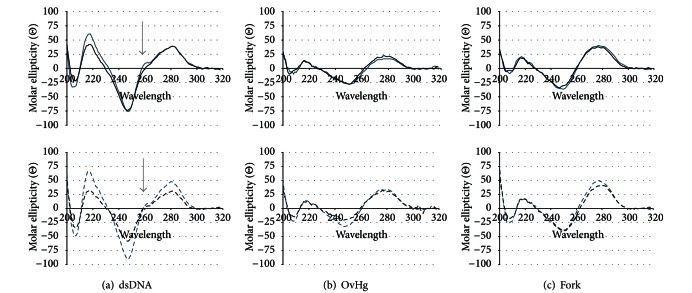
CD spectra comparing the effect of **3** on the secondary structure of replication relevant architectures. The top row shows the CD spectra for constructs of Sequence 1 (solid lines), while the bottom row shows the CD spectra for Sequence 2 (dashed lines). Constructs containing unmodified oligomers are represented in blue, while the constructs containing **3** are in black. (a) duplexes, (b) 5′ and 3′ OvHgs, (c) fork.

To determine the effect of **3** on the overall conformation of these substrates, CD analysis of duplex, 5′ and 3′-OvHgs and fork DNA architectures containing **3** was performed (Figure 4). Previously, it was observed that **3** has a minimal effect on the secondary structure of duplex DNA [10]. All architectures, with and without **3**, were observed to be predominately B-form as indicated by the presence of a positive band around 280 nm and a negative band around 245 nm [25]. Thus, the presence of **3** does not have a significant effect on the global conformation of these substrates. In comparing the architectures for Sequence 1 to those of Sequence 2, more pronounced differences between 2′-deoxyribose versus **3** containing architectures of Sequence 2 were observed as indicated by decreases in the minima at 245 nm and maxima at 280 nm. In duplex substrates **2C** and **2D**, the presence of **3** causes a decrease in the intensity of the minima at 245 nm and the maxima at 280 nm. In the 3′-OvHg **2B**, the presence of **3** significantly decreases the intensity of the minimum at 245 nm, but has minimal to no affect on the maximum at 280 nm. Additionally, fork **2F** shows no change at the minimum while a decrease in the maximum is observed. Taken together, the presence of **3** at a single location has more of an effect on base pairing interactions in duplex DNA, OvHg, and fork architectures of Sequence 2, than Sequence 1.

 In considering the architectures for Sequence 1, the comparisons between those containing unmodified oligomers and those containing **3**, 5′-OvHg **1A** and **1B** and fork **1E** and **1F**, indicate no significant difference in overall conformation. Interestingly, the region between 240 and 300 nm in the spectra was obtained for duplexes **1C** and **1D**; there are also minimal differences with the exception of the shoulder at 260 nm. In the presence of **3**, this shoulder is lost. This is an important observation as the presence of this shoulder is characteristic of poly A•T duplex regions [26]. However, this shoulder is not lost in the analysis of the duplex containing **3** of Sequence 2 (**2D**). This indicates that poly A•T base pairing in duplex DNA is affected by the presence of **3** in Sequence 1, but not in Sequence 2, suggesting a sequence dependent effect on duplex structure by the presence of a single **3**. 

### 3.4. Summary: Affects of **3** on Structure and Stability of Replication Relevant Architectures

Thermal melting and CD analysis are powerful methods for the evaluation of changes in DNA stability and structure. Here we demonstrate differences in substrate stability and structure introduced by the presence of **3** in duplex, OvHg and fork architectures. This is the first time that 5′-OvHg, 3′-OvHg, and fork constructs containing **3** have been reported. These results show that **3** destabilizes all DNA architectures investigated. It appears that **3** has a greater effect when located closer to the 3′ end of the strand. In Sequence 1, where **3** is located near the 5′ end of the strand, the *T*_*m*_ and CD analysis of 5′-OvHgs **1A** and **1B** and forks **1E** and **1F **show no change in stability and insignificant differences in conformation. This indicates that **3** does not disrupt base pairing in the duplex region 3′ of **3**. In the case of 3′-OvHg **2B** and fork **2F**, where **3 **is located near the 3′ end of the oligonucleotide, both decreases in stability and conformational changes are evident, indicating that **3** effects base pairing 5′ of the lesion. In duplex DNA, the presence of **3** causes the characteristic poly A•T shoulder to be lost in the CD spectra of **1D**, but maintained in **2D**. If **3** affects base-pairing interactions in both the 5′ and 3′ directions, it is expected that both duplex constructs of Sequences 1 and 2 containing **3** would lose the poly A•T shoulder in the CD spectra. Provided that there are only five base pairs 5′ of **3** in **1D**, and twelve base pairs 5′ of **3** in **2D**, the differences in the duplex CD spectra can be attributed to an effect of **3** on conformation towards the 5′ end, resulting in a greater disruption in the structure of Sequence 1. Interestingly, despite the conformation differences indicated through CD analysis between unmodified and **3** containing sequences, both duplex constructs containing **3** are destabilized to the same extent. Given that base context, the position of **3** and design of the architecture are conserved between Sequence 1 and 2, and the observed changes can only be attributed to **3**. 

### 3.5. Melting Temperature Analysis of Architectures Containing Overhangs and Phosphates

Strand-breaks resulting from 2′-deoxyribose oxidation are often associated with phosphorylated 3′ and 5′ ends. The effects of such lesions on the stability of model replication forks for both Sequence 1 and 2 were determined (Table 2). The presence of a 5′ or 3′ terminal phosphate on duplexes **1H** and **2H** decreases the *T*_*m*_ values by 0.4°C and 0.9°C, respectively. Previously, it was shown that changes in *T*_*m*_ caused by a 3′ or 5′ terminal phosphate on duplex DNA are dependent on counter-ion concentration [18]. At comparable counter ion concentrations, Bower et al. observed a decrease in *T*_*m*_ in the presence of a 5′ or 3′ phosphate by 0.8°C and 0.3°C, respectively [18]. Besides the difference in experimental design, our experiments have the terminal phosphate residing beside an A•T bp, while in the work by Bower et al. it resides beside a G•C bp. The small difference in destabilization observed between the 5′ and 3′ phosphates herein and those reported by Bower et al. suggests that the terminal base-pair adjacent to the phosphate may contribute to the effects of the terminal phosphates.

The presence of a 6 mer 3′-OvHg (**1I **& **1 K**) decreases the *T*_*m*_ of the core duplex by 1.9–2.8°C, while a 5′-OvHg (**2I** & **2 K**) causes a less significant decrease of 1.1–1.3°C. It has been widely demonstrated that overhangs consisting of one nucleotide, regardless of polarity, are stabilizing [20]. This stabilization is attributed to base stacking between the unpaired nucleoside and the neighboring base pair [22]. Overhangs consisting of two nucleotides have also been found to increase stability [19]. Our results indicate that the presence of either a 6 mer 3′ or 5′-OvHg decreases core duplex stability. Interestingly, the range in Δ*T*_*m*_ for the 5′-OvHgs is significantly smaller than that of the 3′-OvHgs. This suggests that the base context of the single-stranded region influences the *T*_*m*_ of the 3′-OvHg more than the 5′-OvHg. Additional studies using 3 mer, 4 mer, and 5 mer OvHgs will be pursued to further evaluate the effect of overhang length on *T*_*m*_.

In using the constructs with a phosphate at the ss-dsDNA junction, both the effect of the overhang and phosphate were evaluated to determine how their combined presence influences core duplex stability. Compared to the Δ*T*_*m*_ introduced by the OvHgs alone, we observed that a 3′-OvHg decreases the *T*_*m*_ by an additional 0.6°C when a phosphate is present at the ss-dsDNA junction **(1J **& **1L**), while the 5′-OvHg decreases duplex stability by an additional 0.3°C in the presence of a phosphate (**2J **& **2L**). As seen previously in the case of OvHgs without a phosphate, the range of destabilization introduced by the 5′-OvHgs with a phosphate at the ss-dsDNA junction is lower than that of the comparable 3′-OvHgs. This further suggests that the decrease in stability is influenced by the base context of the 3′-OvHg region, but not by the 5′-OvHg region. Additionally, the decrease in *T*_*m*_ introduced by the 5′ phosphate at the ss-dsDNA junction (**1J** & **1L**) is more than twice that observed for the terminal 5′ phosphate (**1H**), while the 3′ phosphate at the ss-dsDNA junction (**2J** & **2L**) has only a slightly greater effect on *T*_*m*_ than the terminal 3′ phosphate (**2H**). Together, these results indicate that the 3′-OvHg and 3′ phosphate have a larger effect on the stability of the duplex region than the corresponding 5′-OvHg and 5′ phosphate. Furthermore, decreases in duplex stability are observed when both an overhang and phosphate are present. 

### 3.6. DSC Analysis of Architectures Containing Overhangs and Phosphates

Differential scanning calorimetry of C3′-thymidinyl radical derived single strand-break products of the model replication fork was performed to thermodynamically characterize the effects of OvHgs and phosphates on the core duplex DNA as indicated in Table 3. Previously, using dangling ends, the presence of a 5′ phosphate was shown to decrease the Δ*G* of formation for duplex DNA, while the 3′ phosphate introduces minimal to no change [18]. Additionally, favorable changes in Δ*G* of formation and melting have been observed for both 3′ and 5′ dangling ends up to two dangling bases, indicating stabilization [19–22]. Interestingly, dangling ends ranging from 3 to 10 bases have been reported to be stabilizing in the case of the 5′ end and destabilizing in the case of the 3′ end [23]. Our results indicate that both the 5′ and 3′-OvHgs are destabilizing. The presence of a 3′-OvHg destabilizes by an average of 0.93 kcal/mol, while the 5′-OvHg destabilizes by 0.16 kcal/mol. The presence of a single 3′ or 5′ terminal phosphate also causes destabilization of the duplex. These results show no measurable difference in ΔΔ*G*_25_ between 3′ versus 5′ terminal phosphates, indicating that the presence of a terminal phosphate, increases the Δ*G*_25_ by an average of +0.38 kcal/mol. Additionally, the destabilization introduced by a 3′-OvHg in the presence of the phosphate at the ss-dsDNA junction compared to the 5′-OvHg in the presence of the phosphate at the same position resulted in no significant difference in ΔΔ*G*_25_ for constructs **1L** and **2L**. Alternatively, in constructs **1J** and **2J**, the 3′-OvHg with the phosphate at the ss-dsDNA junction has no effect on DNA stability, while the comparable 5′-OvHg destabilizes by 0.80 kcal/mol. Lastly, the effect introduced by the phosphate at the ss-dsDNA junction of overhangs is found to be destabilizing in the case of the 3′ phosphate in **2J **and **2L** (+0.82 kcal/mol), stabilizing in the case of the 5′ phosphate in **1J** (−0.76 kcal/mol) and not significant in **1L**. Together, these results show that all strand-break products, consisting of an individual overhang, phosphate, or both, contain less stable duplexes for both Sequence 1 and 2, as indicated by unfavorable ΔΔ*G*_25_ values in all cases except for **1J**. 

### 3.7. Summary: Effect of Overhangs and Phosphates on the Stability of Replication Relevant Architectures

 As previously mentioned, all architectures investigated, except for **1J**, are less stable as indicated by the Δ*T*_*m*_ and ΔΔ*G*_25_ values for UV and DSC analyses. Here one can determine the effects of an individual 3′ or 5′ phosphate and an individual 3′ or 5′-OvHg. Both UV melting and DSC results indicate that 3′-OvHgs are more destabilizing than 5′-OvHgs. 3′-OvHgs are more destabilizing and lower the *T*_*m*_ by ~1°C more than the 5′-OvHgs. Additionally, 3′-OvHgs (**1I **& **1 K**) demonstrate an increase in both entropy and enthalpy, while 5′ OvHg **2 K** demonstrates a decrease in entropy and enthalpy. In the case of the 3′-OvHg, the observed destabilization is enthalpic in nature, while in the case of the 5′-OvHg, destabilization is entropic in nature. Thus, based on the small sampling of DNA OvHg constructs investigated here, these results indicate that both a single 3′- and 5′-OvHg of six nucleotides are destabilizing, but the 3′-OvHg is more destabilizing and enthalpy driven, while the 5′-OvHg is entropy driven. 

With respect to the terminal phosphates, both UV melting and DSC analyses indicate that the 3′ phosphate (**2H**) decreases the *T*_*m*_ to a greater extent than the 5′ phosphate (**1H**). By averaging the *T*_*m*_ values of the UV melting and DSC experiments, it is observed that the 3′ phosphate decreases the *T*_*m*_ by 0.70°C, about twice the change induced by the 5′ phosphate, 0.37°C. Interestingly, no measurable difference in Δ*G*_25_ is observed for an individual 3′ or 5′ terminal phosphate, indicating that polarity does not affect the destabilization introduced by a terminal phosphate. The destabilization observed in the presence of a single 3′ or 5′ terminal phosphate is enthalpy driven, thermodynamically following the trend found for the 3′-OvHgs. 

In evaluating the constructs containing both an overhang and phosphate, the destabilization introduced by the overhang region in the presence of a phosphate at the ss-dsDNA junction appears to be heavily dependent on base context and polarity. This is demonstrated by the lack of differences observed in ΔΔ*G*_25_ values for constructs **1L** and **2L**, indicating a 6 mer overhang of mixed base context to be destabilizing to the same extent, regardless of polarity. While in the case of constructs **1J** and **2J**, the 3′-OvHg introduces no measurable change in ΔΔ*G*_25_, and the comparable 5′-OvHg destabilizes by +0.80 kcal/mol. Intriguingly, the effect of the phosphate at the ss-dsDNA junction appears to be sequence dependent as indicated by the destabilization introduced by the 3′ phosphate in **2J** and **2L**, stabilization by the 5′ phosphate in **1J**, and the lack of effect on stability in the case of the 5′ phosphate in** 1L**.

### 3.8. Possible Impact of C3′-Thymidinyl Radical Damage Products on DNA Replication

The formation of **3** in DNA oligomers has not yet been verified *in vivo*. Given the high levels of formation of **3** *in vitro*, there is a strong possibility that this lesion also forms in biological systems [9]. The effects of this lesion on DNA stability and structure, as well as previously reported effects of 1-(2′-deoxy-*β*-D-*threo*-pentofuranosyl)nucleotides on the enzymatic cleavage of the sugar-phosphate backbone [27], indicate that **3** will have a significant impact on genomic integrity if left unrepaired. Thus, the fidelity of replication processes should be investigated to determine the physiological consequences of this lesion. Currently, detailed structural studies investigating the effect of **3** on DNA structure are being pursued to understand fully the structural impact of the presence of this lesion. 

Alternatively, evaluating the implications of 3′ and 5′-OvHgs and phosphates on DNA stability related to replication may provide insight into the thermodynamic contributions of DNA to facilitate protein-DNA binding and selectivity. The counter-ion condensation (CC) theory has been applied to DNA and DNA-protein complexes and found to be reliable in determining the driving forces for DNA-protein interactions [28]. Specifically, it has been determined that the binding energy required for DNA-protein interactions can be divided into electrostatic and nonelectrostatic components [28]. The electrostatic portion is completely entropic in nature, while the nonelectrostatic portion, which is responsible for specificity, corresponds to the binding enthalpy [28]. Given the CC theory and the results reported herein for strand-break product stability, DNA stability has the potential to be utilized to establish the driving force of binding for replication proteins that recognize and bind to these architectures. Two DNA replication relate proteins that may heavily rely on differences in duplex stability in replication relevant architectures are the single-stranded DNA binding proteins (SSBs) and DNA polymerases.

Single-stranded DNA binding proteins, which are generally classified as nonspecific in binding, are essential in both replication and repair processes. These proteins have demonstrated some intriguing preferences for DNA polarity depending on species. The human single-stranded DNA binding protein, known as Replication Protein A (RPA), has been reported to preferentially bind to 3′-OvHgs [29] and demonstrates a directionality of binding with respect to the 3′ and 5′ ends of the bound DNA [30–32]. Additionally, the single-stranded DNA binding protein of *T4* demonstrates a preference for 5′-OvHgs, while that of *E. coli* demonstrates a binding preference for 3′-OvHgs [29]. Given that the destabilization of the 3′-OvHg is found to be enthalpy driven, this may suggest that the hRPA and *E. coli* single-stranded DNA binding proteins preferential bind 3′-OvHgs to form more favorable nonelectrostatic interactions. If this is the case, then the energy barrier to forming these non-electrostatic DNA-protein interactions may be lower in the case of 3′-OvHg, than with 5′-OvHg, as the non-electrostatic component is the driving force of 3′OvHg destabilization. This hypothesis is in good agreement with the literature, of which calorimetric studies of the *E. coli* single-stranded DNA binding protein binding to DNA have been reported to be enthalpically driven [33]. DNA-protein binding is expected to be favorable with respect to both DNA and protein, while the inherent decrease in enthalpy introduced by the 3′-OvHg may increase binding for the *E. coli* single-stranded DNA binding protein by suppressing the energy barrier of binding. 

A wide variety of DNA polymerases exist for replication and repair of DNA. Recently, it was reported that Pol I in *E. coli* (Klenow) preferentially binds to DNA replication architectures as compared to those generated from strand-breaks, suggesting that the Klenow polymerase can selectively associate with DNA replication substrates [34]. Specifically, the presence of a 3′ phosphate at the ss-dsDNA junction was observed to decrease the binding affinity by 0.9–1.5 kcal/mol, depending on the presence of magnesium [34]. Our results indicate that the presence of a 3′ phosphate at the ss-dsDNA junction destabilizes to the extent of 0.82 kcal/mol, suggesting that the presence of the 3′ phosphate and its impact on DNA stability accounts for a significant amount of destabilization in the DNA-protein binding complex. Furthermore, the destabilization introduced by this 3′ phosphate is enthalpy driven, suggesting that the binding of Klenow is likely enthalpy dependent. Previous results reported the binding of Klenow at physiological temperatures to be enthalpy driven [35], being in good agreement with our hypothesis. This further supports the significance of DNA damage lesions and their impact on DNA stability in understanding the formation and stability of DNA-protein interactions. 

## 4. Conclusions

The impact of strand-break products on DNA stability in model DNA architectures mimicking those generated through 2′-deoxyribose oxidation at the C3′-position during replication has been analyzed. The results obtained demonstrate that both an individual 3′ or 5′-OvHg of six nucleotides is destabilizing, with the 3′-OvHg destabilizing to a greater extent than the 5′-OvHg. Also, the presence of an individual 3′ or 5′ terminal phosphate was observed to be destabilizing, but polarity was not found to affect the magnitude of destabilization. Intriguingly, enthalpy is the driving force of destabilization for the 3′-OvHg and both 3′ and 5′ terminal phosphates, while the 5′-OvHg is entropy driven. Alternatively, the effects on substrate stability of OvHgs containing a phosphate at the ss-dsDNA junction are highly dependent on base context and polarity. In addition to these strand-break substrates, we determined the effects of **3** on the structure and stability of dsDNA, fork, 3′ and 5′-OvHg. The presence of **3** was observed to have a greater impact on both stability and structure when located closer to the 3′ end of the oligonucleotide strand. Taken together, evaluating the impact of lesions previously observed as a result of C3′-radical formation in replication relevant architectures provides insight into how these DNA damage lesions alter the stability and structure of replication associated nucleotide substrates, directly expanding the current scope of how oxidatively generated sugar damage impacts DNA integrity. With these results reported herein and our previous results in determining the impact of DNA structure on the fate of the C3′-thymidinyl radical, the role of proteins can now be evaluated to determine the interplay between oxidative sugar damage and protein binding.

## Figures and Tables

**Table 1:** Comparison of  *T*_*m*_ values for unmodified and **3** containing DNA architectures relevant to replication. The position of **3** is indicated by “Z”.

ID	Base Sequence		*T*_*m*_ (°C)	Δ*T*_*m*_ (°C)
**1**	5′-CGCAACCTGAAA_X_			
	3′-GCGTTGGACTTT_Y_			
5′-OvHg				
**1A**	X = OH	Y = TTTTTT	51.6 ± 0.3	—
**1B**	X = OH	Y = ZTTTTT	51.6 ± 0.1	0
dsDNA				
**1C**	X = AAAAAA	Y = TTTTTT	57.9 ± 0.1	—
**1D**	X = AAAAAA	Y = ZTTTTT	52.8 ± 0.1	−5.1
Fork				
**1E**	X = GACTGT	Y = TTTTTT	47.3 ± 0.3	—
**1F**	X = GACTGT	Y = ZTTTTT	47.3 ± 0.2	0

**2**	5′-GCGTTGGACTTT_X_			
	3′-CGCAACCTGAAA_Y_			
3′-OvHg				
**2A**	X = TTTTTT	Y = OH	51.6 ± 0.3	—
**2B**	X = ZTTTTT	Y = OH	50.2 ± 0.3	−1.4
dsDNA				
**2C**	X = TTTTTT	Y = AAAAAA	58.9 ± 0.1	—
**2D**	X = ZTTTTT	Y = AAAAAA	53.6 ± 0.2	−5.3
Fork				
**2E**	X = TTTTTT	Y = GACTGT	48.6 ± 0.2	—
**2F**	X = ZTTTTT	Y = GACTGT	47.9 ± 0.2	−0.7

**Table 2:** Comparison of *T*_*m*_ values for replication relevant DNA architectures containing individual 3′ and 5′ overhangs, a terminal phosphate, and presence of both an overhang and phosphate. The presence of a 3′ or 5′ phosphate (PO_4_), hydroxyl (OH) and a 6 mer overhang is indicated as an “X” or “Y” at the end of the core dsDNA sequences. Δ*T*_*m*_ values were obtained by subtracting the *T*_*m*_ of each construct from the *T*_*m*_ value of the core duplex.

ID	Base Sequence		*T*_*m*_ (°C)	Δ*T*_*m*_ (°C)
**1**	5′-CGCAACCTGAAA_X_			
	3′-GCGTTGGACTTT_Y_			
**1G**	X = OH	Y = OH	51.7 ± 0.2	—
**1H**	X = OH	Y = PO_4_	51.3 ± 0.0	−0.4^a^
**1I**	X = AAAAAA	Y = OH	48.9 ± 0.3	−2.8^b^
**1J**	X = AAAAAA	Y = PO_4_	47.9 ± 0.1	−3.4^b^
				−1.0^a^
**1K**	X = GACTGT	Y = OH	49.8 ± 0.3	−1.9^b^
**1L**	X = GACTGT	Y = PO_4_	48.7 ± 0.2	−2.6^b^
				−1.1^a^

**2**	5′-GCGTTGGACTTT_X_			
	3′-CGCAACCTGAAA_Y_			
**2G**	X = OH	Y = OH	52.9 ± 0.2	—
**2H**	X = PO_4_	Y = OH	52.0 ± 0.2	−0.9^a^
**2I**	X = OH	Y = AAAAAA	51.8 ± 0.1	−1.1^b^
**2J**	X = PO_4_	Y = AAAAAA	50.5 ± 0.3	−1.5^b^
				−1.3^a^
**2K**	X = OH	Y = GACTGT	51.6 ± 0.2	−1.3^b^
**2L**	X = PO_4_	Y = GACTGT	50.4 ± 0.2	−1.6^b^
				−1.2^a^

^a^Indicates the Δ*T*_*m*_ introduced by an individual phosphate. Δ*T*_*m*_ was determined by subtracting substrate G from H, I from J and K from L. ^b^Indicates the Δ*T*_*m*_ introduced by an individual overhang. Δ*T*_*m*_ was determined by subtracting substrate G from I, G from K, H from J, and H from L.

**Table 3:** Thermodynamic parameters obtained from differential scanning calorimetry for replication relevant DNA architectures containing individual 3′ and 5′ overhangs, a terminal phosphate, and presence of both an overhang and phosphate. The presence of a 3′ or 5′ phosphate (PO_4_), hydroxyl (OH) and a 6 mer overhang is indicated as an “X” or “Y” in the core dsDNA sequences.

ID	Base Sequence		*T*_*m*_ (°C)	Δ*H*_DSC_ (kcal/mol)	Δ*H*_vH_ (kcal/mol)	Δ*S* (cal/mol∗K)	Δ*G*_25_ (kcal/mol)	Δ*T*_*m*_ (°C)	ΔΔ*H*_DSC_ (kcal/mol)	ΔΔ*S* (cal/mol∗K)	ΔΔ*G*_25_ (kcal/mol)
**1**	5′-CGCAACCTGAAA_X_										
	3′-GCGTTGGACTTT_Y_										
**1G**	X = OH	Y = OH	60.91 ± 0.10	−77.28 ± 0.82	−62.39 ± 0.29	−231.4 ± 2.5	−8.34 ± 0.07	—	—	—	—
**1H**	X = OH	Y = PO_4_	60.62 ± 0.05	−74.67 ± 0.21	−62.34 ± 0.11	−223.7 ± 0.7	−8.01 ± 0.02	−0.29^a^	+2.61^a^	+7.7^a^	+0.33^a^
**1I**	X = AAAAAA	Y = OH	59.01 ± 0.05	−70.93 ± 0.12	−63.90 ± 0.08	−213.6 ± 0.4	−7.29 ± 0.01	−1.90^b^	+6.35^b^	+17.8^b^	+1.05^b^
**1J**	X = AAAAAA	Y = PO_4_	57.79 ± 0.02	−80.85 ± 0.39	−60.66 ± 0.12	−244.3 ± 1.2	−8.05 ± 0.04	−2.83^b^	−6.18^b^	−20.6^b^	−0.04^b^
								−1.22^a^	−9.92^a^	−30.7^a^	−0.76^a^
**1K**	X = GACTGT	Y = OH	58.72 ± 0.07	−73.83 ± 0.40	−61.58 ± 0.07	−222.5 ± 1.2	−7.53 ± 0.05	−2.19^b^	+3.45^b^	+8.9^b^	+0.81^b^
**1L**	X = GACTGT	Y = PO_4_	58.16 ± 0.03	−74.32 ± 0.73	−62.22 ± 0.36	−224.3 ± 2.2	−7.47 ± 0.07	−2.46^b^	+0.35^b^	−0.6^b^	+0.54^b^
								−0.56^a^	−0.49^a^	−1.8^a^	+0.06^a^
**2**	5′-GCGTTGGACTTT_X_										
	3′-CGCAACCTGAAA_Y_										
**2G**	X = OH	Y = OH	61.88 ± 0.08	−88.31 ± 0.44	−61.38 ± 0.34	−263.6 ± 1.4	−9.76 ± 0.03	—	—	—	—
**2H**	X = PO_4_	Y = OH	61.37 ± 0.05	−86.31 ± 0.65	−59.81 ± 0.36	−258.0 ± 1.9	−9.42 ± 0.08	−0.51^a^	+2.00^a^	+5.6^a^	+0.34^a^
**2I**	X = OH	Y = AAAAAA	61.04 ± 0.02	−88.48 ± 0.36	−68.32 ± 0.11	−264.8 ± 1.1	−9.58 ± 0.04	−0.84^b^	−0.17^b^	−1.2^b^	+0.18^b^
**2J**	X = PO_4_	Y = AAAAAA	60.15 ± 0.13	−81.38 ± 0.15	−68.64 ± 0.16	−244.2 ± 0.4	−8.62 ± 0.04	−1.22^b^	+4.93^b^	+13.8^b^	+0.80^b^
								−0.89^a^	+7.10^a^	+20.6^a^	+0.96^a^
**2K**	X = OH	Y = GACTGT	60.37 ± 0.01	−90.36 ± 0.50	−64.46 ± 0.33	−270.9 ± 1.5	−9.62 ± 0.06	−1.51^b^	−2.05^b^	−7.3^b^	+0.14^b^
**2L**	X = PO_4_	Y = GACTGT	59.88 ± 0.07	−84.91 ± 0.30	−65.66 ± 0.20	−255.0 ± 0.9	−8.93 ± 0.04	−1.49^b^	+1.40^b^	+3.0^b^	+0.49^b^
								−0.49^a^	+5.45^a^	+15.9^a^	+0.69^a^

^a^Indicates the effects introduced by an individual phosphate. ΔΔ*H*_DSC_, ΔΔ*S*, and ΔΔ*G*_25_ were determined by subtracting substrate G from H, I from J, and K from L. ^b^Indicates the effects introduced by an individual overhang. ΔΔ*H*_DSC_, ΔΔ*S*, and ΔΔ*G*_25_ were determined by subtracting substrate G from I, G from K, H from J, and H from L.
